# Tris(2-amino­pyridinium) hexa­chlorido­indate(III)

**DOI:** 10.1107/S1600536811043285

**Published:** 2011-10-29

**Authors:** Xu-Dong Jin, Li-Cai Sun, Hai-Bo Wang, Chun-Hua Ge

**Affiliations:** a College of Chemistry, Liaoning University, Shenyang 110036, People’s Republic of China

## Abstract

The Schiff base (*E*)-4-chloro-2-[(pyridin-2-yl­imino)­meth­yl]phenol was reacted with InCl_3_·4H_2_O, generating the title molecular salt, (C_5_H_7_N_2_)_3_[InCl_6_]. The octa­hedral hexa­chlorido­indate(III) anion is located on an inversion centre, and one half of the anion and two crystallographically independent cations form the asymmetric unit. One of the cations is located on a twofold rotation axis and its intra-ring C and N atoms simulate this symmetry by exchanging their positions in statistical disorder. In the crystal, weak N—H⋯Cl hydrogen bonds and two types of π–π interactions with centroid–centroid separations of 4.047 (3) and 4.202 (3) Å are observed.

## Related literature

For the synthesis of 2-amino­pyridine and salicyl­aldehyde Schiff bases, see: Burlova *et al.*(2008 ▶).
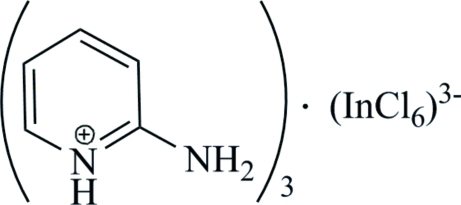


## Experimental

### 

#### Crystal data

(C_5_H_7_N_2_)_3_[InCl_6_]
*M* *_r_* = 612.90
Monoclinic, 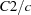

*a* = 18.6491 (17) Å
*b* = 16.2454 (14) Å
*c* = 8.4004 (5) Å
β = 112.214 (1)°
*V* = 2356.1 (3) Å^3^
*Z* = 4
Mo *K*α radiation
μ = 1.70 mm^−1^
*T* = 298 K
0.46 × 0.43 × 0.05 mm

#### Data collection

Bruker SMART CCD area-detector diffractometer
Absorption correction: multi-scan (*SADABS*; Sheldrick, 1996 ▶) *T*_min_ = 0.509, *T*_max_ = 0.925
5734 measured reflections
2053 independent reflections
1672 reflections with *I* > 2σ(*I*)
*R*_int_ = 0.030

#### Refinement

*R*[*F* ^2^ > 2σ(*F* ^2^)] = 0.036
*wR*(*F* ^2^) = 0.100
*S* = 1.08
2053 reflections
130 parameters
H-atom parameters constrained
Δρ_max_ = 0.96 e Å^−3^
Δρ_min_ = −0.79 e Å^−3^

### 

Data collection: *SMART* (Bruker, 2002 ▶); cell refinement: *SAINT* (Bruker, 2002 ▶); data reduction: *SAINT*; program(s) used to solve structure: *SHELXS97* (Sheldrick, 2008 ▶); program(s) used to refine structure: *SHELXL97* (Sheldrick, 2008 ▶); molecular graphics: *SHELXTL/PC* (Sheldrick, 2008 ▶); software used to prepare material for publication: *SHELXTL/PC*.

## Supplementary Material

Crystal structure: contains datablock(s) I, global. DOI: 10.1107/S1600536811043285/kp2354sup1.cif

Structure factors: contains datablock(s) I. DOI: 10.1107/S1600536811043285/kp2354Isup2.hkl

Supplementary material file. DOI: 10.1107/S1600536811043285/kp2354Isup3.cdx

Supplementary material file. DOI: 10.1107/S1600536811043285/kp2354Isup4.mol

Supplementary material file. DOI: 10.1107/S1600536811043285/kp2354Isup5.mol

Additional supplementary materials:  crystallographic information; 3D view; checkCIF report

## Figures and Tables

**Table 1:** Selected bond lengths (Å)

In1—Cl1	2.5121 (11)
In1—Cl2	2.5406 (11)
In1—Cl3	2.5120 (12)

**Table 2:** Hydrogen-bond geometry (Å, °)

*D*—H⋯*A*	*D*—H	H⋯*A*	*D*⋯*A*	*D*—H⋯*A*
N1—H1⋯Cl2^i^	0.86	2.43	3.242	158
N2—H2*A*⋯Cl2^i^	0.86	2.58	3.354	150
N2—H2*B*⋯Cl1^ii^	0.86	2.75	3.558	157
N2—H2*B*⋯Cl3^ii^	0.86	2.82	3.359	122
N3—H3a⋯Cl3^iii^	0.86	2.70	3.549	169
N3—H3a⋯Cl2^iii^	0.86	2.91	3.337	112
N4—H4*A*⋯Cl1	0.86	2.56	3.358	154

Symmetry codes: (i) 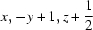; (ii) 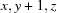; (iii) 
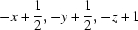
.

**Fig. 1.**
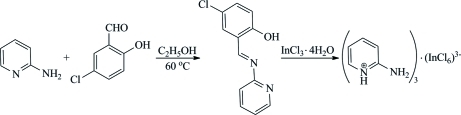
The chemical reaction scheme.

**Fig. 2.**
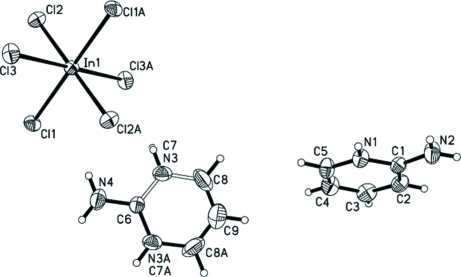
ORTEP drawing of the title compound with the atomic numbering scheme. Displacement ellipsoids are drawn at the 30% probability level. H atoms are drawn as small spheres of arbitrary radii. The atoms C7/N3 are statistically disordered exchanging location (50:50) to simulate the twofold rotation symmetry.

**Fig. 3.**
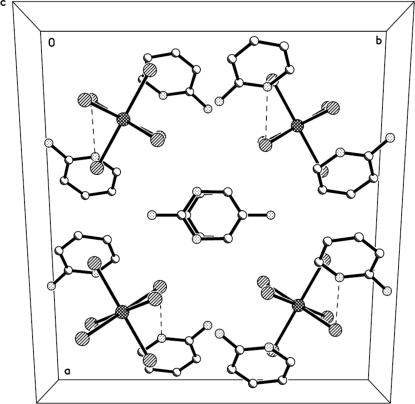
A view of a packing section of the title compound, stacking along the c axis. Dashed lines indicate intramolecular hydrogen bonds.

## supplementary crystallographic information

### Comment

Instead of an expected formation of a Schiff base indium complex, the reaction of the Schiff base with InCl_3_.4H_2_O in anhydrous alcohol leads to the title compound (Fig. 1). It could be reasonably explained that the Schiff base molecule decomposed in presence of water when a reactant InCl_3_.4H_2_O in alcohol was heated at 333 K.

A single-crystal analysis reveals that the asymmetric unit of the complex comprises a half of a hexachloro-indium anion and two 2-amino pyridinium cations. The indium (III) ion is located at the inversion centre (the special position 1/4, 1/4, 1/2 in the space group C2/c) surrounded by six Cl atoms in the octahedral coordination (Table 1, Fig. 2). The three crystallographically independent In—Cl bond lengths [2.5120 (12), 2.5121 (11) and 2.5406 (11) Å] (Table 1, Fig. 2) characterise the coordination. One of pyridinium cations involves the two C atoms (C6 and C9) located at the twofold axis whereas the atoms C8, C7 and N3 are in general position (Fig. 2). However, C7 and N3 (pp = 1/2) are in statistical disorder to simulate the twofold symmetry. The N1—C5 cation is in general position. The pyridinium cations (one in general position and one in a special position - totally three cations per structural unit) balance the charge of InCl_6_^3-^. An interplanar angle between two pyridine rings of N1/C1–C5 and N3/C6–C9 is 67.37°. A pair of weak π–π stacking interactions are found between the almost parallel pyridine rings, N1/C1–C5 and its symmetry related moiety (symmetry code: -*x* + 1, *y*, -*z* + 3/2) with the centroid separation of 4.047 (3) Å, perpendicular distance of 3.647 (2) Å and an angle of 9°. In addition, another pair of π–π stacking interactions (with amino groups in staggered arrangement in stacked rings) is observed in the crystal packing between the antiparallel aromatic rings, N3/C6–C9 and its symmetry related ring (symmetry code: -*x* + 1, -*y* + 1, -*z* + 1), with the interplanar spacing of 3.847 (3) Å, slippage of 1.690 Å and the centroid separation of 4.202 (3) Å. In the crystal packing, looking down the *c* axis, a cavity can be seen (Fig. 3).

### Experimental

In this work, the Schiff base was prepared according to the similar method (Burlova *et al.*, 2008). A mixture of 5-chlorosalicylaldehyde (0.470 g, 3.0 mmol) and 2-aminopyridine (0.285 g, 3.0 mmol) in 12 ml anhydrous alcohol was stirred at 333 K for 2 h, a yellow Schiff base precipitate was filtered and dried. Then a solution of Schiff base (0.235 g, 1.0 mmol) in 10 ml anhydrous alcohol was added to another solution of InCl_3_.4H_2_O (0.293 g, 1.0 mmol) in 3 ml of anhydrous alcohol. The mixture was stirred at 333 K for *ca* 2.5 h, concentrated and left to stand at room temperature. Yellow single crystals suitable for X-ray analysis were obtained by slow solvent evaporation in 10 d.

### Refinement

H atoms attached to N atoms were located in a difference Fourier synthesis and allowed to refine with a fixed isotropic displacement parameter of *U*iso(H) = 1.2*U*eq(N), distance restraint of N—H = 0.86 Å. All other H atoms were constrained to idealized geometries (C—H = 0.98 Å) and were assigned isotropic displacement parameters of *U*iso(H) = 1.2*U*eq(C).

### Crystal data

**Table:**

(C_5_H_7_N_2_)_3_[InCl_6_]	*F*(000) = 1216
*M**_r_* = 612.90	*D*_x_ = 1.728 Mg m^−^^3^
Monoclinic, *C*2/*c*	Melting point: 465.0 K
Hall symbol: -C 2yc	Mo *K*α radiation, λ = 0.71073 Å
*a* = 18.6491 (17) Å	Cell parameters from 3465 reflections
*b* = 16.2454 (14) Å	θ = 2.5–28.3°
*c* = 8.4004 (5) Å	µ = 1.70 mm^−^^1^
β = 112.214 (1)°	*T* = 298 K
*V* = 2356.1 (3) Å^3^	Plate, yellow
*Z* = 4	0.46 × 0.43 × 0.05 mm

### Data collection

**Table:**

Bruker SMART CCD area-detector diffractometer	2053 independent reflections
Radiation source: fine-focus sealed tube	1672 reflections with *I* > 2σ(*I*)
graphite	*R*_int_ = 0.030
φ and ω scans	θ_max_ = 25.0°, θ_min_ = 3.5°
Absorption correction: multi-scan (*SADABS*; Sheldrick, 1996)	*h* = −22→14
*T*_min_ = 0.509, *T*_max_ = 0.925	*k* = −19→19
5734 measured reflections	*l* = −9→9

### Refinement

**Table:**

Refinement on *F*^2^	Primary atom site location: structure-invariant direct methods
Least-squares matrix: full	Secondary atom site location: difference Fourier map
*R*[*F*^2^ > 2σ(*F*^2^)] = 0.036	Hydrogen site location: inferred from neighbouring sites
*wR*(*F*^2^) = 0.100	H-atom parameters constrained
*S* = 1.08	*w* = 1/[σ^2^(*F*_o_^2^) + (0.0466*P*)^2^ + 6.8482*P*] where *P* = (*F*_o_^2^ + 2*F*_c_^2^)/3
2053 reflections	(Δ/σ)_max_ = 0.001
130 parameters	Δρ_max_ = 0.96 e Å^−^^3^
0 restraints	Δρ_min_ = −0.79 e Å^−^^3^

### Special details

**Table:**

Geometry. All e.s.d.'s (except the e.s.d. in the dihedral angle between two l.s. planes) are estimated using the full covariance matrix. The cell e.s.d.'s are taken into account individually in the estimation of e.s.d.'s in distances, angles and torsion angles; correlations between e.s.d.'s in cell parameters are only used when they are defined by crystal symmetry. An approximate (isotropic) treatment of cell e.s.d.'s is used for estimating e.s.d.'s involving l.s. planes.
Refinement. Refinement of *F*^2^ against ALL reflections. The weighted *R*-factor *wR* and goodness of fit *S* are based on *F*^2^, conventional *R*-factors *R* are based on *F*, with *F* set to zero for negative *F*^2^. The threshold expression of *F*^2^ > σ(*F*^2^) is used only for calculating *R*-factors(gt) *etc*. and is not relevant to the choice of reflections for refinement. *R*-factors based on *F*^2^ are statistically about twice as large as those based on *F*, and *R*-factors based on ALL data will be even larger.

### Fractional atomic coordinates and isotropic or equivalent isotropic displacement parameters (Å^2^)

**Table:**

	*x*	*y*	*z*	*U*_iso_*/*U*_eq_	Occ. (<1)
In1	0.2500	0.2500	0.5000	0.03637 (17)	
Cl1	0.37640 (6)	0.17532 (7)	0.57322 (15)	0.0487 (3)	
Cl2	0.19049 (7)	0.15088 (7)	0.25064 (15)	0.0490 (3)	
Cl3	0.20941 (7)	0.15470 (7)	0.68614 (16)	0.0529 (3)	
N1	0.3380 (2)	0.8489 (2)	0.6354 (5)	0.0473 (9)	
H1	0.3042	0.8353	0.6775	0.057*	
N2	0.3012 (3)	0.9833 (3)	0.6428 (7)	0.0728 (14)	
H2A	0.2669	0.9670	0.6813	0.087*	
H2B	0.3061	1.0349	0.6263	0.087*	
N3	0.4384 (3)	0.4542 (3)	0.6425 (7)	0.0632 (13)	0.50
H3	0.3987	0.4285	0.5728	0.076*	0.50
N4	0.5000	0.3304 (4)	0.7500	0.083 (2)	
H4A	0.4605	0.3039	0.6810	0.099*	
C1	0.3466 (3)	0.9288 (3)	0.6088 (6)	0.0471 (11)	
C2	0.4025 (3)	0.9494 (3)	0.5426 (7)	0.0593 (13)	
H2	0.4109	1.0042	0.5228	0.071*	
C3	0.4442 (3)	0.8897 (4)	0.5076 (7)	0.0645 (15)	
H3A	0.4812	0.9037	0.4630	0.077*	
C4	0.4331 (3)	0.8075 (4)	0.5366 (7)	0.0653 (15)	
H4	0.4623	0.7665	0.5126	0.078*	
C5	0.3792 (3)	0.7888 (3)	0.6000 (7)	0.0581 (13)	
H5	0.3702	0.7341	0.6195	0.070*	
C6	0.5000	0.4118 (4)	0.7500	0.0477 (16)	
C7	0.4384 (3)	0.4542 (3)	0.6425 (7)	0.0632 (13)	0.50
H7	0.3954	0.4264	0.5671	0.076*	0.50
C8	0.4397 (5)	0.5396 (5)	0.6454 (11)	0.103 (3)	
H8	0.3972	0.5688	0.5719	0.123*	
C9	0.5000	0.5795 (7)	0.7500	0.117 (4)	
H9	0.5000	0.6368	0.7500	0.140*	

### Atomic displacement parameters (Å^2^)

**Table:**

	*U*^11^	*U*^22^	*U*^33^	*U*^12^	*U*^13^	*U*^23^
In1	0.0320 (3)	0.0386 (3)	0.0448 (3)	0.00200 (17)	0.02156 (19)	−0.00423 (18)
Cl1	0.0379 (6)	0.0538 (7)	0.0584 (7)	0.0112 (5)	0.0227 (5)	−0.0016 (5)
Cl2	0.0460 (6)	0.0546 (7)	0.0533 (7)	−0.0071 (5)	0.0265 (5)	−0.0144 (5)
Cl3	0.0598 (7)	0.0535 (7)	0.0597 (7)	0.0051 (6)	0.0388 (6)	0.0072 (5)
N1	0.055 (2)	0.049 (2)	0.052 (2)	0.0054 (19)	0.0357 (19)	0.0002 (18)
N2	0.076 (3)	0.047 (2)	0.108 (4)	−0.001 (2)	0.050 (3)	0.006 (3)
N3	0.039 (3)	0.075 (3)	0.069 (3)	−0.005 (2)	0.013 (2)	0.004 (3)
N4	0.054 (4)	0.045 (4)	0.156 (8)	0.000	0.047 (4)	0.000
C1	0.051 (3)	0.046 (3)	0.047 (3)	0.004 (2)	0.021 (2)	0.004 (2)
C2	0.060 (3)	0.056 (3)	0.068 (3)	0.017 (3)	0.031 (3)	0.000 (3)
C3	0.061 (3)	0.079 (4)	0.070 (4)	0.013 (3)	0.043 (3)	0.000 (3)
C4	0.068 (4)	0.068 (4)	0.076 (4)	−0.004 (3)	0.046 (3)	0.009 (3)
C5	0.074 (4)	0.047 (3)	0.069 (3)	−0.003 (3)	0.044 (3)	0.000 (3)
C6	0.043 (4)	0.044 (4)	0.065 (4)	0.000	0.030 (3)	0.000
C7	0.039 (3)	0.075 (3)	0.069 (3)	−0.005 (2)	0.013 (2)	0.004 (3)
C8	0.086 (5)	0.086 (5)	0.120 (6)	0.033 (4)	0.020 (5)	0.042 (5)
C9	0.122 (11)	0.065 (6)	0.155 (12)	0.000	0.043 (9)	0.000

### Geometric parameters (Å, °)

**Table:**

In1—Cl1^i^	2.5121 (11)	N4—H4A	0.8600
In1—Cl1	2.5121 (11)	C1—C2	1.395 (7)
In1—Cl2^i^	2.5406 (11)	C2—C3	1.344 (7)
In1—Cl2	2.5406 (11)	C2—H2	0.9300
In1—Cl3^i^	2.5120 (12)	C3—C4	1.386 (8)
In1—Cl3	2.5120 (12)	C3—H3A	0.9300
N1—C1	1.336 (6)	C4—C5	1.339 (7)
N1—C5	1.344 (6)	C4—H4	0.9300
N1—H1	0.8600	C5—H5	0.9300
N2—C1	1.330 (6)	C6—C7^ii^	1.351 (6)
N2—H2A	0.8600	C6—N3^ii^	1.351 (6)
N2—H2B	0.8600	C8—C9	1.307 (10)
N3—C6	1.351 (6)	C8—H8	0.9300
N3—C8	1.387 (9)	C9—C8^ii^	1.307 (10)
N3—H3	0.8600	C9—H9	0.9300
N4—C6	1.322 (9)		
			
Cl3^i^—In1—Cl3	180.00 (4)	N2—C1—C2	123.9 (5)
Cl3^i^—In1—Cl1^i^	91.47 (4)	N1—C1—C2	117.1 (5)
Cl3—In1—Cl1^i^	88.53 (4)	C3—C2—C1	119.6 (5)
Cl3^i^—In1—Cl1	88.53 (4)	C3—C2—H2	120.2
Cl3—In1—Cl1	91.47 (4)	C1—C2—H2	120.2
Cl1^i^—In1—Cl1	180.0	C2—C3—C4	121.3 (5)
Cl3^i^—In1—Cl2^i^	88.97 (4)	C2—C3—H3A	119.3
Cl3—In1—Cl2^i^	91.03 (4)	C4—C3—H3A	119.3
Cl1^i^—In1—Cl2^i^	88.44 (4)	C5—C4—C3	118.3 (6)
Cl1—In1—Cl2^i^	91.56 (4)	C5—C4—H4	120.9
Cl3^i^—In1—Cl2	91.03 (4)	C3—C4—H4	120.9
Cl3—In1—Cl2	88.97 (4)	C4—C5—N1	120.1 (5)
Cl1^i^—In1—Cl2	91.56 (4)	C4—C5—H5	120.0
Cl1—In1—Cl2	88.44 (4)	N1—C5—H5	120.0
Cl2^i^—In1—Cl2	180.00 (4)	N4—C6—C7^ii^	120.7 (3)
C1—N1—C5	123.6 (4)	N4—C6—N3^ii^	120.7 (3)
C1—N1—H1	118.2	C7^ii^—C6—N3^ii^	0.0 (7)
C5—N1—H1	118.2	N4—C6—N3	120.7 (3)
C1—N2—H2A	120.0	C7^ii^—C6—N3	118.7 (6)
C1—N2—H2B	120.0	N3^ii^—C6—N3	118.7 (6)
H2A—N2—H2B	120.0	C9—C8—N3	120.9 (7)
C6—N3—C8	119.6 (5)	C9—C8—H8	119.6
C6—N3—H3	120.2	N3—C8—H8	119.6
C8—N3—H3	120.2	C8—C9—C8^ii^	120.5 (11)
C6—N4—H4A	120.0	C8—C9—H9	119.8
N2—C1—N1	118.9 (5)	C8^ii^—C9—H9	119.8
			
C5—N1—C1—N2	177.8 (5)	C1—N1—C5—C4	1.0 (8)
C5—N1—C1—C2	−0.9 (7)	C8—N3—C6—N4	179.8 (5)
N2—C1—C2—C3	−178.1 (5)	C8—N3—C6—C7^ii^	−0.2 (5)
N1—C1—C2—C3	0.5 (8)	C8—N3—C6—N3^ii^	−0.2 (5)
C1—C2—C3—C4	−0.3 (9)	C6—N3—C8—C9	0.4 (11)
C2—C3—C4—C5	0.3 (9)	N3—C8—C9—C8^ii^	−0.2 (5)
C3—C4—C5—N1	−0.6 (9)		

Symmetry codes: (i) −*x*+1/2, −*y*+1/2, −*z*+1; (ii) −*x*+1, *y*, −*z*+3/2.

### Hydrogen-bond geometry (Å, °)

**Table:**

*D*—H···*A*	*D*—H	H···*A*	*D*···*A*	*D*—H···*A*
N1—H1···Cl2^iii^	0.86	2.43	3.242	158.
N2—H2A···Cl2^iii^	0.86	2.58	3.354	150.
N2—H2B···Cl1^iv^	0.86	2.75	3.558	157.
N2—H2B···Cl3^iv^	0.86	2.82	3.359	122.
N3—H3a···Cl3^i^	0.860	2.702	3.549	168.53
N3—H3a···Cl2^i^	0.860	2.914	3.337	112.30
N4—H4A···Cl1	0.86	2.56	3.358	154.

Symmetry codes: (iii) *x*, −*y*+1, *z*+1/2; (iv) *x*, *y*+1, *z*; (i) −*x*+1/2, −*y*+1/2, −*z*+1.

## References

[bb1] Bruker (2002). *SMART* and *SAINT* Bruker AXS Inc., Madison, Wisconsin, USA.

[bb2] Burlova, A. S., Uraeva, A. I. & Ikorskiib, V. N. (2008). *Russ. J. Gen. Chem.* **7**, 1230–1235.

[bb3] Sheldrick, G. M. (1996). *SADABS* University of Göttingen, Germany.

[bb4] Sheldrick, G. M. (2008). *Acta Cryst.* A**64**, 112–122. 10.1107/S0108767307043930 18156677

